# Acknowledgement to Reviewers of *Genes* in 2014

**DOI:** 10.3390/genes6010022

**Published:** 2015-01-07

**Authors:** Genes Editorial Office

**Affiliations:** MDPI AG, Klybeckstrasse 64, CH-4057 Basel, Switzerland

The editors of *Genes* would like to express their sincere gratitude to the following reviewers for assessing manuscripts in 2014:

Al-Kateb, Hussam
Angrist, Misha
Anney, Richard
Aparicio, Ana
Auer, Pauk
Azziz, Ricardo
B. Avraham, Karen
Barrett, Angela N.
Bartlett, Christopher W.
Berger, Michael F.
Bergmann, Jan H.
Beumer, Kelly J.
Bianchetti, Laurent
Binder, Elisabeth
Blanton, Susan
Boardman, Lisa
Bocquet, Béatrice
Brait, Mariana
Bull, Susan
Calderari, Sophie
Carrell, Douglas T.
Cassa, Christopher A.
Chan, Dylan K.
Chang, Qiang
Chen, Chongyi
Chiu, Charles
Cho, Raymond
Cho, William
Chung, Wendy K.
Cole, Charles N.
Craig, David W.
Das, Samarjit
Davie, Jim
de Bakker, Paul I.W.
De, Subhajyoti
Demers, Jill
Derringer, Jaime
Doetsch, Paul
Dove, Edward S.
Downing, Nancy R.
Dryga, Anatoly
Duncan, Laramie
Elisabetta, Donzelli
Ellard, Sian
Faas, Brigitte
Fazio, Pietro Di
Fehse, Boris
Fiszer, Agnieszka
Fox, Archa
Francisci, Silvia
Frayling, Timothy
Fusco, Alfredo
Gonzalez, Roser
Grant, Julia D.
Gregory, Philip A.
Gribble, Matthew
Gruen, Jeffrey R.
Grundberg, Elin
Grünwald, David
Gruppuso, Philip A.
Hall, Deborah A.
Hall, Michael J.
Hammond, Christopher J.
Harris, Reuben S.
Haubold, Bernhard
Hawi, Ziarih
Hemminki, Kari
Hertz, DL
Hiesinger, Robin
Hilakivi-Clarke, Leena
Hinney, Anke
Hussain, Khalid
Irimia, Manuel
Isaacs, Aaron
Kamiya, Hideki
Kamsteeg, Erik-Jan
Kantharidis, Phillip
Kato, Akihiko
Kipling, David
Knoppers, Bartha M.
Kolialexi, Aggeliki
Kono, Michihiro
Krampis, Konstantinos
Kuiper, Roland
Le, Thuc
Lee, Ji-Eun
Lee, Sang Kil
Lettre, Guillaume
Li, Bingshan
Li, Jun Z.
Lindblom, Annika
Linsday, Howard
Liu, Fan
Liu, Li
Lohmueller, Kirk
Lu, Zhixiang
Lynch, Henry T.
Maddox, Paul
Madhyastha, R
Maier, André
Majewski, Jacek
Manchia, Mirko
Marceau, Kristine
Martin-Leo, Hansmann
McCormick, Jennifer B
Meglecz, Emese
Mendez, Juan
Meyer, Urs
Montpetit, Ben
Müller-Myhsok, Bertram
Murillas, Rodolfo
Naujok, Ortwin
Offenbacher, Steven
Ogino, Shuji
Ophoff, Roel
Ozcan, Sabire
Palancade, Benoit
Palmero, Ignacio
Pandya, Arti
Park, Mi Kyoung
Parker, Lisa
Pavlov, Youri I.
Pendergrass, Sarah A.
Peyrard-Janvid, Myriam
Pierotti, Marco Alessandro
Ponchel, F
Poretti, Andrea
Povirk, Lawrence F.
Resendes, Karen K.
Ribeiro, Andre S.
Rout, Michael P.
Roy, Somak
Ruppert, Volker
Rutschmann, Sophie
Sacchetti, Lucia
Safe, Stephen
Sahoo, Satya S
Sakrikar, Dhananjay
Sandford, Richard
Sayan, Emre
Schaid, Daniel
Schulze-Bahr, Eric
Sen, Aritro
Sharma, Manu
Shen, Yiping
Siggs, Owen M.
Silvestri, Gabriella
Sleiman, Patrick M.A.
Smith, Steven
Song, Kyuyoung
Soranzo, Nicole
Spencer, David
Stankiewicz, Pawel
Strauss, Jerome F.
Tamborero, David
Tilgner, Hagen
Tomasetti, Marco
Tomblin, Bruce
Tost, Heike
Travis, William
Tremblay, Jacques
Trono, Didier
Tsai, Kuo-Wang
Tyson, Frederick L.
Usheva, Anny
Vance, Russell E.
Vecoli, Cecilia
Vegvari, Akos
Vicentini, Caterina
Viel, Alessandra
Vodicka, Pavel
von Arnim, Albrecht
Walter, Jörn
Wang, Xiaonan
Wei, Zong
Weir, Tiffany L.
Wickramasinghe, Vihandha O.
Wildonger, Jill
Yan, Shan
Yen, Yun
Zhang, Qin
zhang, Wei
Zhao, Fengqi

